# Supplementary material for: Neurological events and unanticipated risks after locoregional anesthesia (NEURAL): Protocol for a multicenter prospective observational study
Source: PLoS One. 2026 May 5;21(5):e0348493. doi: 10.1371/journal.pone.0348493 (PMC13143103; doi:10.1371/journal.pone.0348493)
Supplement: S2 File — (DOCX) [file pone.0348493.s002.docx]

**Appendix 1**

**Neurological Events and Unanticipated Risks After Locoregional Anesthesia (NEURAL): Protocol for a Multicenter Prospective Observational Study**

**Short title: NEURAL Protocol**

Alessandro De Cassai, Elena Ioppolo, Dario Bugada, Francesco Tasso, Gianluca Cappelleri, Vito Torrano

**Telephone Questionnaire**

Good morning Mr./Ms. ____________, this is Dr. ___________. I am calling you regarding the NEURAL study, which aims to investigate possible issues resulting from regional anesthesia, for which you gave consent to participate on ________, at the time of your ___________ surgery.

Since the day before the surgery, have you noticed any of the following changes?

1. **Do you feel you have reduced sensation in the ___________ (sensory area consistent with the anesthesia performed)?** YES/NO
    If yes, what do you feel less?
   - Touch sensation  YES NO
   - Warmth      YES NO
   - Pain       YES NO
2. **Do you feel you are less able to move the area that was operated on?** YES/NO
    If yes, please describe the type of deficit (e.g., partial/complete deficit in foot extension):
3. **Pain** Do you have pain? (in the area consistent with the nerve block)

If yes, DN4 questionnaire

DN4>4 = chronic pain
