# Supplementary material for: Neurological events and unanticipated risks after locoregional anesthesia (NEURAL): Protocol for a multicenter prospective observational study
Source: PLoS One. 2026 May 5;21(5):e0348493. doi: 10.1371/journal.pone.0348493 (PMC13143103; doi:10.1371/journal.pone.0348493)
Supplement: S3 File — (DOCX) [file pone.0348493.s003.docx]

**Appendix 2**

**Neurological Events and Unanticipated Risks After Locoregional Anesthesia (NEURAL): Protocol for a Multicenter Prospective Observational Study**

**Short title: NEURAL Protocol**

Alessandro De Cassai, Elena Ioppolo, Dario Bugada, Francesco Tasso, Gianluca Cappelleri, Vito Torrano

English Protocol

**Introduction** Regional anesthesia has an extremely high safety profile [1]. However, complications related to regional techniques are reported in the literature (e.g., local anesthetic toxicity, hematoma requiring medical attention, nerve injury) [2–4].

The incidence of these complications is not fully known. The main reason is that the incidence is determined by registry studies, often with limited sample sizes.

Furthermore, while for some complications the cause-effect mechanism of regional anesthesia is clear (for example, a pneumothorax following a paravertebral block), for other complications (such as nerve injury after regional anesthesia), the mechanism of action is unclear.

Historically, nerve injury was thought to result from direct needle contact with nerve fibers, i.e., essentially mechanical trauma. However, recent evidence casts doubt on this concept, showing that it is mechanically difficult to cause nerve injury and that nerve injuries can occur even without direct contact with the nerve. Therefore, alongside other causal factors such as local anesthetic toxicity, sub-perineural injection, high injection pressures under the epineurium, and sub-epineural hematoma after forced needle-nerve contact, altered coagulation profiles, intrinsic patient fragility, comorbidities, or other currently unknown factors may play a role in the pathogenesis of nerve injury [5].

**Study Objective**

**Primary objective:** To determine the incidence of complications after regional anesthesia (aggregated outcome: nerve injury, hematoma, pneumothorax, local anesthetic systemic toxicity), calculated as the number of events (“complication”: nerve injury, hematoma, pneumothorax, local anesthetic toxicity) divided by the total number of procedures.

**Secondary objectives:**

- Determine the overall and individual incidence of complications for each upper limb block (outcomes considered individually: nerve injury, hematoma, pneumothorax, local anesthetic systemic toxicity), calculated as the number of events divided by the total number of procedures.
- Determine the overall and individual incidence of complications for each lower limb block (outcomes considered individually: nerve injury, hematoma, local anesthetic systemic toxicity), calculated as the number of events divided by the total number of procedures.
- Determine the overall and individual incidence of complications for each fascial block (outcomes considered individually: nerve injury, hematoma, pneumothorax, local anesthetic systemic toxicity), calculated as the number of events divided by the total number of procedures.
- Identify risk factors for complications following upper limb regional anesthesia.
- Identify risk factors for specific complications following lower limb regional anesthesia.
- Identify risk factors for complications following fascial block.

**Definitions** *Note: For all complication definitions, the conditions are considered to have occurred after the procedure and not pre-existing.*

- **Hematoma:** Collection of blood at the regional anesthesia site requiring medical and/or surgical intervention.
- **Pneumothorax:** Evidence of air consistent with the diagnosis of pneumothorax on the same side as the regional anesthesia, confirmed by ultrasound, chest X-ray, or CT scan.
- **Local anesthetic systemic toxicity (LAST):** Any ECG, hemodynamic, and/or neurological alteration following regional anesthesia attributed to absorption of local anesthetic requiring treatment (e.g., lipid emulsion therapy).
- **Nerve injury:** Onset of dysesthesia, anesthesia, or prolonged motor deficit not explained by the pharmacokinetics of the anesthetic used, occurring in the area corresponding to the block.

**Regional anesthesia of the upper limb:** Using the latest nomenclature of the European Society of Regional Anesthesia [6]. Blocks considered: Interscalene brachial plexus block; Superior trunk block; Supraclavicular brachial plexus block; Infraclavicular brachial plexus block (various approaches); Suprascapular nerve block (anterior/posterior); Axillary brachial plexus block; Superficial, intermediate, and deep cervical plexus blocks.

**Regional anesthesia of the lower limb:** Using the latest nomenclature of the European Society of Regional Anesthesia [6]. Blocks considered: Lumbar plexus block; Sacral plexus block; Fascia iliaca block (supra-/infra-inguinal approaches); Adductor canal block; PENG block; Femoral nerve block; Sciatic nerve block (various approaches); Nerve to vastus medialis block; Genicular nerves block; IPACK; Ankle block; Pudendal nerve block.

**Fascial regional anesthesia:** Using the latest nomenclature of the European Society of Regional Anesthesia [7]. Blocks considered: Rectus sheath block; Ilioinguinal-iliohypogastric block; TAP blocks; ESP block; SAP block; PIP block; IPP block; TFP block; Rhomboid intercostal block; Retrolaminar block; Midaxillary TAP block; Quadratus lumborum blocks (anterior, lateral, posterior); Paravertebral block; Intertransverse process block; Pectoserratus block.

**Study Design** Prospective multicenter observational study.

**Inclusion and Exclusion Criteria** **Population:** Adult patients (≥18 years) undergoing single-shot regional anesthesia of the upper limb, lower limb, or fascial block.

**Inclusion criteria:**

- Age ≥18 years
- Single-shot plexus/fascial anesthesia
- Informed consent

**Exclusion criteria:**

- More than one single-shot plexus/fascial anesthesia in the same body region
- Continuous plexus/fascial anesthesia
- Language barrier preventing follow-up

**Participating centers:** The study aims to recruit as many centers as possible, subject to local Ethics Committee approval. Recruitment is non-competitive; the list of centers may be updated progressively. A current list of participating centers will be sent semiannually to the Ethics Committee of the coordinating center (University Hospital of Padua), including the names and contacts of newly authorized centers.

**Methods** Upon obtaining informed consent, the following data will be collected using a standardized data collection form:

**Intraoperative data:**

- Demographics: age, sex, BMI
- Clinical history: relevant comorbidities (hypertension, diabetes, peripheral/central neuropathy)
- Medications: especially anticoagulants and antiplatelets, with dosages
- Procedural data: pre-procedural sedation (agent and dose, sedation level via RASS), injection pain (yes/no), type of regional anesthesia, use of ultrasound (yes/no, visualization of structures, in-plane/out-of-plane approach, nerve swelling), nerve stimulator (max/min intensity), pressure limiter, anesthetic used (volume and mg dose), single anesthetic vs mixture, use of adjuvants (type and dose)

**Postoperative data:**

- Presence (yes/no) of hematoma or nerve deficits (sensory: dysesthesia, anesthesia, paresthesia; motor deficits) at 24 and 48 hours.

*Note:* Because nerve deficits may not be immediately apparent or may be masked by factors such as local edema or limb immobilization, sensory and motor deficits will also be evaluated at 15 and 30 days via phone interview using a standardized questionnaire (see Appendix 1). Patients reporting neurological deficits will be referred and managed according to local protocols. Follow-up calls will occur every 30 days until resolution, up to a maximum of one year.

**Summary of follow-up:** Contact at 24h, 48h, 15d, 30d; for nerve complications, treatment per local hospital protocol and follow-up every 30 days until resolution or one year.

**Statistical Analysis**

- Continuous variables: normality assessed with Shapiro-Wilk test.
- Normally distributed: mean ± SD; non-normal: median and IQR.
- Comparisons: Student’s t-test (normal), Mann-Whitney U (non-normal); categorical: χ² or Fisher’s exact test.
- Correlation: Pearson (normal), Spearman (non-normal).
- Logistic regression to assess associations between categorical dependent variables (e.g., complications) and independent predictors; odds ratios with 95% CI. Multicollinearity assessed via variance inflation factors.
- Analyses performed using R; p<0.05 considered significant.

**Sample Size Calculation** Observational study investigating very rare complications; incidence in the literature ranges from 0–2.8% [8]. Sample size estimated using Agresti-Coull Wilson method [9], starting with a cumulative incidence of 0.5% [10], 95% confidence, and CI width of 0.003 → 3396 patients required.

The NEURAL study anticipates at least 10 ICUs participating, with ~350 patients per center over two years, making the target sample size feasible.

**Study Duration:** Two years; each center recruits for six months after local ethics approval.

**Regulatory and Ethical Considerations**

- Study conducted according to the Declaration of Helsinki.
- Written informed consent required.
- Compliance with GDPR (EU 2016/679) and Italian clinical trial regulations (D.lgs. 211/2003).

**Data Management:**

- Data collected via REDCap®, pseudo-anonymized before entry.
- Secure storage, encrypted connections, hierarchical access control.
- Data retained for five years, then anonymized.
- Subsequent use for scientific purposes authorized by study sponsor and steering committee, in compliance with GDPR.

**Ownership and Publication:**

- Data belong to participating centers; SIAARTI is co-owner.
- Steering Committee supervises statistical analysis and manuscript preparation.
- Local coordinators ensure protocol adherence.
- Authorship recognized under "SIAARTI Study Group" rules (one investigator per 50 enrolled patients).

**Patient Risk:**

- No additional risk; procedures are routine.
- No special insurance coverage required due to observational nature.

**Conclusion:** This ambitious multicenter observational study will provide valuable data on the incidence of postoperative complications in patients undergoing regional anesthesia.

Original language (Italian)

**Introduzione** L’anestesia loco-regionale presenta un profilo di sicurezza estremamente elevato [1]. Tuttavia complicanze legate alle tecniche locoregionali sono riportate in letteratura (ad esempio tossicità da anestetico locale, ematoma che richiede attenzione medica, danno nervoso)[2-4].

L’incidenza di tali complicanze non è del tutto nota, il motivo principale è che l’incidenza è determinata da studi su registro spesso su una numerosità campionaria limitata.

Inoltre,mentre per alcune complicanze il meccanismo causa-effetto dell’anestesia locoregionale è lampante (si pensi ad esempio a un pneumotorace conseguente a un blocco paravertebrale) per altre complicanze (quali ad esempio il danno nervoso dopo anestesia locoregionale) il meccanismo di azione non è chiaro.

Se infatti storicamente si pensava che il danno nervoso fosse dovuto al contatto diretto dell’ago con le fibre nervose, e quindi a un traumatismo essenzialmente meccanico, recenti evidenze mettono in dubbio tale concetto dimostrando come sia meccanicamente difficile provocare un danno nervoso e come danni nervosi possano ben avvenire anche senza contatto diretto con il nervo. Pertanto insieme ad altri fattori causali quali tossicità dell'anestetico locale, l'iniezione sotto-perineurale, le alte pressioni di iniezione sotto-epineurale del fluido e l'ematoma sotto-epineurale dopo un contatto forzato tra ago e nervo, un profilo coagulativo alterato e altri fattori quali la fragilità intrinseca del paziente, eventuali comorbidità, o altri fattori attualmente sconosciuti possono svolgere un ruolo nella patogenesi del danno.[5]

**Obiettivo dello Studio**

**Obiettivo primario dello studio:** è determinare l’incidenza delle complicanze dopo anestesia loco-regionale (Outcome aggregato: Danno nervoso, ematoma, pneumotorace, sindrome da tossicità da anestetico locale) calcolato come numero di eventi “complicanza (danno nervoso, ematoma, pneumotorace, sindrome da tossicità da anestetico locale)” su numero totale di procedure.

**Obiettivi secondari dello studio:**

- Determinare l’incidenza di complicanze globale e per ciascun blocco dell’arto superiore (Considerati singolarmente i seguenti outcome: Danno nervoso, ematoma, pneumotorace, sindrome da tossicità da anestetico locale), calcolato come numero di eventi su numero totale di procedure.
- Determinare l’incidenza di complicanze globale e per ciascun blocco dell’arto inferiore (Considerati singolarmente i seguenti outcome: Danno nervoso, ematoma, sindrome da tossicità da anestetico locale), calcolato come numero di eventi su numero totale di procedure.
- Determinare l’incidenza di complicanze globale e per ciascun blocco fasciale (Considerati singolarmente i seguenti outcome: Danno nervoso, ematoma, pneumotorace, sindrome da tossicità da anestetico locale), calcolato come numero di eventi su numero totale di procedure.
- Determinare i fattori di rischio per l’insorgenza delle complicanze dopo anestesia loco-regionale dell’arto superiore.
- Determinare i fattori di rischio per l’insorgenza delle diverse complicanze dopo anestesia loco-regionale dell’arto inferiore.
- Determinare i fattori di rischio per l’insorgenza delle complicanze dopo esecuzione di blocco fasciale.

**Definizioni** *NB: Per tutte le definizioni di complicanze le condizioni si intendono insorte dopo la procedura e non pre-esistenti prima della procedura*

- **Ematoma:** raccolta ematica in sede in procedura locoregionale che richiede intervento medico e/o chirurgico.
- **Pneumotorace:** Evidenza di falce aerea compatibile con diagnosi di pneumotorace omolaterale alla sede di anestesia locoregionale, obiettivata tramite ecografia, rx torace o tc torace.
- **Sindrome da tossicità da anestetico locale:** qualsiasi alterazione elettrocardiografica, emodinamica e/o neurologica successiva all’anestesia locoregionale attribuita al riassorbimento di anestetico locale con conseguente trattamento (es. Somministrazione di miscela lipidica).
- **Danno nervoso:** Insorgenza di disestesia, anestesia, o deficit motorio prolungato non spiegabile dalla farmacocinetica dell’anestetico utilizzato in territorio congruo con l’anestesia effettuata.

**Anestesia locoregionale arto superiore:** Verrà utilizzata la nomenclatura più recente dell’European Society of Regional Anesthesia [6]. I blocchi considerati saranno: Interscalene brachial plexus block; Superior trunk block; Supraclavicular brachial plexus block; Infraclavicular brachial plexus block (coracoid, retroclavicular, costoclavicular approaches); Suprascapular nerve block (anterior/posterior approach); Axillary brachial plexus block; Superficial, Intermediate, Deep cervical plexus blocks.

**Anestesia locoregionale arto inferiore:** Verrà utilizzata la nomenclatura più recente dell’European Society of Regional Anesthesia [6]. I blocchi considerati saranno: Lumbar plexus block; Sacral plexus block; Fascia iliaca block (supra-/infra-inguinal approach); Adductor canal block; Pericapsular nerve group (PENG) block; Femoral nerve block; Femoral triangle block; Sciatic nerve block (anterior, transgluteal, infragluteal, popliteal); Nerve to vastus medialis block; Genicular nerves block; IPACK; Common peroneal nerve block; Ankle block; Pudendal nerve block.

**Anestesia locoregionale fasciale:** Verrà utilizzata la nomenclatura più recente dell’European Society of Regional Anesthesia [7]. I blocchi considerati saranno: Rectus sheath block; Ilioinguinal iliohypogastric nerves block; TAP block; Subcostal TAP block; Erector spinae plane (ESP) block; Deep serratus anterior plane (SAP) block; Superficial parasternal intercostal plane (PIP) block; Interpectoral plane (IPP) block; Transversalis fascia plane (TFP) block; Rhomboid intercostal plane block; Retrolaminar block; Midaxillary TAP block; Quadratus lumborum blocks (anterior, lateral, posterior); Paravertebral block; Intertransverse process (ITP) block; Superficial serratus anterior plane (SAP) block; Deep parasternal intercostal plane (PIP) block; Pectoserratus plane (PSP) block.

**Disegno dello studio** Studio osservazionale multicentrico prospettico.

**Criteri di inclusione ed esclusione** **Popolazione dello Studio:** Pazienti adulti (≥18 anni) sottoposti ad anestesia locoregionale di arto superiore, inferiore o fasciale single-shot.

**Criteri di Inclusione:**

- Età ≥18 anni
- Programma di anestesia plessica/fasciale single shot
- Consenso informato

**Criteri di Esclusione:**

- Esecuzione di più di una tecnica di anestesia plessica/fasciale single shot nel medesimo distretto corporeo
- Esecuzione di anestesie plessiche fasciale continua
- Barriera linguistica per cui lo sperimentatore reputi impossibile proseguire il follow-up

**Centri coinvolti:** Lo studio prevede l’arruolamento del maggior numero possibile di centri, previa approvazione da parte dei rispettivi Comitati Etici. L’arruolamento non è di tipo competitivo, pertanto la lista dei centri partecipanti è suscettibile di aggiornamenti progressivi nel corso dello studio. Oltre ai centri dello Steering Committee, elencati a pagina 1 del presente protocollo, verrà predisposto e mantenuto un elenco aggiornato dei centri partecipanti. Tale elenco verrà trasmesso con cadenza semestrale al Comitato Etico del centro promotore (Azienda Ospedale – Università di Padova), unitamente ai nominativi e ai riferimenti dei nuovi centri autorizzati a partecipare allo studio.

**Metodi** Previa raccolta del consenso informato si procederà alla raccolta dei seguenti dati su scheda raccolta dati standardizzata:

**Tempo Intraoperatorio:**

- Dati demografici: età, sesso, BMI
- Storia clinica: comorbidità di rilievo (ipertensione arteriosa, diabete mellito, neuropatia periferica, neuropatia centrale)
- Anamnesi farmacologica: attenzione a anticoagulanti, antiaggreganti e dosaggio
- Dati operatori: sedazione pre-procedura (farmaco, dosaggio, livello RASS), dolore all’iniezione si/no, tipo di anestesia locoregionale, utilizzo ecografo (si/no, visualizzazione strutture si/no, approccio in-plane/out-of-plane, swelling nervoso si/no), elettrostimolatore (intensità max/min), limitatore di pressione, anestetico utilizzato (volume e dose mg), singolo anestetico vs miscela, uso adiuvanti (tipo/dosaggio mg/mcg)

**Tempo Postoperatorio:**

- Presenza (si/no) di ematoma o deficit nervoso (si/no), quali (sensitivi: disestesia, anestesia, parestesia; motori) a 24 e 48 ore.

**Nota bene:** I deficit nervosi saranno anche indagati a 15 e 30 giorni tramite colloquio telefonico con questionario standardizzato. I pazienti con deficit neurologici saranno gestiti secondo le consuetudini locali; follow-up telefonico ogni 30 giorni fino a risoluzione, max 1 anno.

**Riassunto follow-up:** 24h, 48h, 15d, 30d.

**Analisi Statistica**

- Variabili continue: Shapiro-Wilk per normalità; normale: media±DS; non normale: mediana e IQR.
- Confronti: t-test (normale), Mann-Whitney (non normale); categoriali: χ² o Fisher.
- Correlazioni: Pearson (normale), Spearman (non normale).
- Regressione logistica per variabili dipendenti categoriali (complicanze), calcolo OR con 95% CI, verifica multicollinearità tramite VIF.
- Analisi con R; p<0.05 significativo.

**Calcolo della Numerosità Campionaria** Incidenza complicanze molto rare: 0–2,8% [8]. Metodo Agresti-Coull Wilson [9]; incidenza cumulativa 0,5% [10]; CI 0,003 → almeno 3396 pazienti.
 Coinvolgimento realistico di 10 centri ICUs, ~350 pazienti per centro in 2 anni.

**Durata dello studio:** Due anni; reclutamento per 6 mesi per ciascun centro.

**Riferimenti normativi e considerazioni etico-amministrative**

- Studio secondo Dichiarazione di Helsinki.
- Consenso informato scritto obbligatorio.
- Conformità GDPR (UE 2016/679) e D.lgs. 211/2003.

**Gestione dati:**

- Raccolta tramite REDCap®, pseudoanonimizzazione, crittografia HTTPS, accesso gerarchico.
- Conservazione 5 anni, poi anonimizzazione.
- Uso successivo solo per scopi scientifici approvati.

**Proprietà e pubblicazione:**

- Dati di proprietà dei centri; SIAARTI co-titolare.
- Steering Committee supervisiona analisi e manoscritto.
- Coordinatori locali garantiscono aderenza al protocollo.
- Autori riconosciuti sotto “Gruppo di Studio SIAARTI” (1 per 50 pazienti).

**Rischi per il paziente:**

- Nessun rischio aggiuntivo; procedure routinarie.
- Nessuna assicurazione speciale necessaria.

**Conclusioni:** Lo studio osservazionale multicentrico permetterà di ottenere dati sull’incidenza di complicanze postoperatorie nei pazienti sottoposti ad anestesia locoregionale.

REFERENCE

1. Neal JM, Barrington MJ, Brull R, Hadzic A, Hebl JR, Horlocker TT, Huntoon MA, Kopp SL, Rathmell JP, Watson JC. The Second ASRA Practice Advisory on Neurologic Complications Associated With Regional Anesthesia and Pain Medicine: Executive Summary 2015. Reg Anesth Pain Med. 2015 Sep-Oct;40(5):401-30. doi: 10.1097/AAP.0000000000000286. PMID: 26288034.
2. Long B, Chavez S, Gottlieb M, Montrief T, Brady WJ. Local anesthetic systemic toxicity: A narrative review for emergency clinicians. Am J Emerg Med. 2022 Sep;59:42-48. doi: 10.1016/j.ajem.2022.06.017. Epub 2022 Jun 13. PMID: 35777259.
3. Gitman M, Fettiplace MR, Weinberg GL, Neal JM, Barrington MJ. Local Anesthetic Systemic Toxicity: A Narrative Literature Review and Clinical Update on Prevention, Diagnosis, and Management. Plast Reconstr Surg. 2019 Sep;144(3):783-795. doi: 10.1097/PRS.0000000000005989. PMID: 31461049.
4. Hewson DW, Bedforth NM, Hardman JG. Peripheral nerve injury arising in anaesthesia practice. Anaesthesia. 2018 Jan;73 Suppl 1:51-60. doi: 10.1111/anae.14140. PMID: 29313904.
5. McLeod GA, Sadler A, Hales TG. Traumatic needle damage to nerves during regional anesthesia: presentation of a novel mechanotransduction hypothesis. Reg Anesth Pain Med. 2022 Jul 25:rapm-2022-103583. doi: 10.1136/rapm-2022-103583. Epub ahead of print. PMID: 35878962.
6. El-Boghdadly K, Albrecht E, Wolmarans M, Mariano ER, Kopp S, Perlas A, et al (2023) Standardizing nomenclature in regional anesthesia: an ASRA-ESRA Delphi consensus study of upper and lower limb nerve blocks. Reg Anesth Pain Med. doi:10.1136/rapm-2023-104884 (In Press)
7. El-Boghdadly K, Wolmarans M, Stengel AD, Albrecht E, Chin KJ, Elsharkawy H, et al (2021) Standardizing nomenclature in regional anesthesia: an ASRA-ESRA Delphi consensus study of abdominal wall, paraspinal, and chest wall blocks. Reg Anesth Pain Med. 46:571-580. doi:10.1136/rapm-2020-102451
8. Neal JM, Barrington MJ, Brull R, Hadzic A, Hebl JR, Horlocker TT, Huntoon MA, Kopp SL, Rathmell JP, Watson JC. The Second ASRA Practice Advisory on Neurologic Complications Associated With Regional Anesthesia and Pain Medicine: Executive Summary 2015. Reg Anesth Pain Med. 2015 Sep-Oct;40(5):401-30. doi: 10.1097/AAP.0000000000000286. PMID: 26288034.
9. Wilson EB. Probable Inference, the Law of Succession, and Statistical Inference. Journal of the American Statistical Association. 1927;22:209–12.
10. Bergman BD, Hebl JR, Ken J, Horlocker TT. Neurologic complications of 405 consecutive continuous axillary catheters. Anesth Analg. 2003;96: 247–252
11. Dorai-Raj S. binom [Internet]. 2022 [cited 9 June 2024]. Retrieved from: https://CRAN.R-project.org/package=binom
