# Supplementary material for: Neurological events and unanticipated risks after locoregional anesthesia (NEURAL): Protocol for a multicenter prospective observational study
Source: PLoS One. 2026 May 5;21(5):e0348493. doi: 10.1371/journal.pone.0348493 (PMC13143103; doi:10.1371/journal.pone.0348493)
Supplement: Attachment — Submitted filename: Reviewiers.docx [file pone.0348493.s005.docx]

EDITORIAL DECISION

**Q1:This is a well-written and engaging manuscript that addresses an important topic. The study is clearly structured, and the findings are presented in a coherent manner. After careful evaluation of the reviewers’ comments and a thorough assessment of the overall quality, originality, and relevance of the submission, the editorial decision is: MINOR REVISION.**

**The reviewers have provided constructive suggestions aimed primarily at improving clarity, presentation, and minor methodological or interpretative aspects. Please find attached reviewer’s comments:**

A1: Dear Editor, thank you for your kind comments. We have tried to respond to Reviewers comments and we believe the overall quality of the manuscript has improved. We are open to further modifications.

REVIEWER 1 – MINOR REVISION

**Q1:The NEURAL study is a large, multicenter prospective observational project designed to determine the true incidence of complications—such as nerve injury, hematoma, pneumothorax, and LAST—following single-shot regional anesthesia. It will enroll over 3,396 adult patients across more than 40 Italian centers, with follow-up extending up to one year for persistent neurological deficits. The study collects detailed patient, procedural, and follow up data to identify both overall incidence and modifiable risk factors. Ultimately, its findings aim to improve patient safety, refine clinical practices, and support updated evidence based guidelines.**

A1: We would like to thank the Reviewer for his time and dedication in improving our manuscript

Minor revisions:

**Q1. Lack of methods to account for clustering**

**The protocol does not address the implications of enrolling patients across more than 40 centers, where outcomes may be correlated within institutions due to shared practices, operator skill, equipment, or workflow. Without incorporating statistical methods that account for clustered data—such as multilevel (mixed effects) models, generalized estimating equations (GEE), or random effects logistic regression—standard errors may be underestimated, leading to inflated Type I error rates. Clarifying how center level variation will be handled is essential.**

A1: We agree with the comment. We have added the following paragraph:”Accounting for Center-Level Clustering

Given the multicenter design of the NEURAL study, patients are clustered within participating institutions, and outcomes may be correlated due to shared clinical practices, operator expertise, equipment, or local protocols. To account for this hierarchical structure, multilevel (mixed-effects) regression models will be used for the primary and secondary analyses. Specifically, random-intercept logistic regression models will be applied to account for between-center variability when estimating associations between predictors and complications. The intraclass correlation coefficient (ICC) will be calculated to quantify the degree of clustering. As a sensitivity analysis, generalized estimating equations (GEE) with robust standard errors will also be considered.”

**Q2. No stated strategy for handling missing data**

**Given the extended follow up period (up to one year for neurological deficits), loss to follow up and incomplete covariate data are likely. The protocol does not describe any approach for addressing missingness, such as multiple imputation, inverse probability weighting, or sensitivity analyses. A predefined plan is necessary to ensure unbiased estimates and transparent reporting.**

A2: Missed data will be handled as shown below:

“If the proportion of missing data exceeds 5% for key variables, multiple imputation by chained equations will be performed under the assumption of missing at random. Imputation models will include all variables used in the primary analyses, including outcomes and relevant predictors. Sensitivity analyses will be conducted to compare complete-case analyses with imputed datasets. In addition, if differential loss to follow-up is observed, inverse probability weighting methods may be applied to assess the robustness of the findings. The amount of missing data and the methods used to address it will be transparently reported according to STROBE recommendations.”

**Q3. Absence of multiple comparison considerations**

**With numerous block types, predictors, and secondary outcomes, the probability of false positive findings increases. Although it is reasonable for exploratory observational studies to avoid overly conservative adjustments, the protocol should acknowledge this issue and clarify whether procedures such as Bonferroni correction, false discovery rate control, or hierarchical testing will be considered in interpreting results.**

A3: We agree, added the following to the method section:

“Given the exploratory nature of this large observational study and the evaluation of multiple block types, predictors, and secondary outcomes, we recognize the potential risk of false-positive findings due to multiple comparisons. The secondary outcomes and subgroup analyses, effect sizes and 95% confidence intervals will be emphasized rather than sole reliance on p-values. Where appropriate, false discovery rate control using the Benjamini-Hochberg procedure may be applied to secondary analyses to reduce the risk of Type I error inflation. All secondary analyses will be interpreted cautiously and considered hypothesis-generating.

“

**Q4. Rare event logistic regression concerns**

**The expected incidence of complications (~0.5%) presents challenges for conventional logistic regression, which can yield biased or unstable estimates when events are sparse. The protocol does not discuss alternative methods tailored to rare events, such as Firth penalized likelihood or exact logistic regression, which may improve model performance and inferential reliability.**

A4: We agree, and we have modified the statistical section that now reads:”

To determine the relationships between the dependent categorical variable (e.g., complications) and one or more independent categorical variables (i.e., complication predictors), logistic regression will be performed to calculate odds ratios (OR) with 95% confidence intervals (CI). Given the anticipated low incidence of complications, rare-event bias may affect conventional maximum likelihood estimation. Therefore, penalized likelihood approaches—such as Firth’s bias-reduced logistic regression—will be used when appropriate to improve the stability and validity of parameter estimates. In cases of extremely sparse data, exact logistic regression methods may also be considered. Model adequacy will be assessed using goodness-of-fit statistics and calibration plots. The presence of multicollinearity will be assessed using variance inflation factors.”

REVIEWER 2 – ACCEPT

**Q1:Dear Authors!**

**Seems to be a well planed study. I don't say it is absolute necessary, but worth thinking to incorporate the early signs of local anaesthetic systemic toxictiy (or is it included in neurological alteration?) and whether they needed intervention or not.**

A1: Dear Reviewer, thank you for the time you spent reading our manuscript. Early signs of toxicity are already incorporated in neurological alteration and have been defined as each symptoms that the treating physician recognized as local anesthetic systemic toxicity
